# Economic cost of tobacco-related cancers in Sri Lanka

**DOI:** 10.1136/tobaccocontrol-2017-053791

**Published:** 2017-10-27

**Authors:** Hemantha Amarasinghe, Sajeeva Ranaweera, Thushara Ranasinghe, Nadeeka Chandraratne, Dinesh Ruwan Kumara, Montarat Thavorncharoensap, Palitha Abeykoon, Amala de Silva

**Affiliations:** 1 Ministry of Health, Nutrition and Indigenous Medicine, Institute of Oral Health, Maharagama, Sri Lanka; 2 Sri Lanka Medical Association, Colombo, Sri Lanka; 3 World Health Organization, Colombo, Sri Lanka; 4 Ministry of Health, Nutrition and Indigenous Medicine, National Authority on Tobacco and Alcohol, Battaramulla, Sri Lanka; 5 Department of Pharmacy, Faculty of Pharmacy, Mahidol University, Bangkok, Thailand; 6 University of Colombo, Colombo, Sri Lanka

**Keywords:** economics, public policy, smoking caused disease, non-cigarette tobacco products

## Abstract

**Introduction:**

Cancer has a high mortality rate and morbidity burden in Sri Lanka. This study estimated the economic cost of smoking and smokeless tobacco (ST) related to cancers in Sri Lanka in 2015.

**Methods:**

Prevalence-based cost of illness is calculated according to the guidelines of the WHO (2011). The direct costs are costs of curative care (costs of inward patients and outpatient care borne by the state and out of pocket expenditure by households) for tobacco-related cancers, weighted by the attributable fractions for these cancers. Indirect costs are lost earnings due to mortality and morbidity (absenteeism of both patient and carers resulting from seeking care and recuperation).

Data were obtained from the Registrar General’s Department, National Cancer Registry, Department of Census and Statistics and the Central Bank of Sri Lanka. Household and systemic costs and relative risks were extracted from research studies. Oncologists (working in both public and private sectors), other clinical specialists, medical administrators and economists were consulted during the estimation and validation processes.

**Results:**

The total economic cost of tobacco-related cancers for Sri Lanka in 2015 was estimated to be US$121.2 million. The direct cost of smoking and ST-related cancers was US$42.1 million, which was 35% of the total cost, while the indirect cost was US$79.1 million, which was 65% of the total cost.

**Conclusion:**

Burden of tobacco smoking and ST-related cancers as reflected in these economic costs is enormous: affecting the healthcare system and country’s economy. Policymakers should take note of this burden and address tobacco consumption control as a priority.

## Introduction

Tobacco is identified as the leading preventable cause of premature death worldwide. It were estimated that 6 million people died from tobacco-related illness in 2014, of which 70% occur from low-income and middle-income countries.^1 2^

This study estimates economic costs of tobacco-related cancers in Sri Lanka and does not estimate the economic costs of other non-communicable diseases related to tobacco such as heart disease, cerebrovascular diseases and lung diseases or communicable diseases which contribute significantly to the burden of tobacco-related diseases.

According to the National Cancer Registry of Sri Lanka, one in every 10 Sri Lankans carries a lifetime risk of developing cancer. The incidence of cancer in Sri Lanka, standardised to the world standard population in the year 2009, was 86.4 and 89.1 per 100 000 populations in males and females, respectively.^3^ Smoking and smokeless tobacco (ST)-related cancers of lip, oral cavity and pharynx, lung, oesophagus and colorectal cancer were the most common cancers among males. Among females, breast and cervix uteri cancers were the most common.^3^ A large body of published epidemiological studies shows strong association between tobacco smoking, ST use and development of cancer. Cancers of the lung, lip, oral cavity and pharynx, oesophagus, larynx, stomach, kidney, pancreas, liver, bladder, colon and rectum are related to smoking,^4^ while cancer of the lip, oral cavity, pharynx and oesophagus are related to ST use.^5 6^ Studies on economic impact of smoking and ST-related cancers have not been previously carried out in Sri Lanka. Similar studies in other low-income and middle-income countries are too scarce. This study was conducted by National Authority on Tobacco and Alcohol, in collaboration with the Expert Committee on Tobacco, Alcohol and Illicit Drugs of the Sri Lanka Medical Association, WHO Country Office and the South East Asian Regional Office, with support from the Faculty of Pharmacy, Mahidol University and the Health Intervention and Technology Assessment Programme, Thailand. The findings of this study emphasises the necessity and the urgency for effective measures to address use of tobacco as a means of preventing cancer in Sri Lanka and the potential economic benefits of such initiatives.

### Prevalence of tobacco smoking and ST use in Sri Lanka

Tobacco is consumed in both smoked and non-smoked or smokeless forms. In Sri Lanka, the most widespread smoked form of tobacco is cigarettes, followed by bidi. Chewing and sniffing are the most common methods of using ST. WHO STEPwise approach to chronic disease risk factor surveillance (STEPS) of 2015 showed that 29.4% males and 0.1% females were current smokers in Sri Lanka, ST use was found in 26% of males and 5% of females.^7^

The WHO Global Youth Tobacco Survey 2015 shows that 3.2% of boys and 0.2% of girls between 13 and 15 years of age smoked at least once during the 30 days preceding the survey in Sri Lanka. The prevalence of ST use among students of this age group was 4.2% among boys and 0.5% among girls.^8^ A study conducted among those over 30 years of age in the villages and estates of the Sabaragamuwa Province of Sri Lanka showed that 53.7% of the study population chewed betel daily; 27%of them were ever smokers.^9^

## Methods

This study was conducted using the guideline ‘Economics of tobacco toolkit: assessment of the economic costs of smoking’ published by the WHO in 2011.^10^ Accordingly, the prevalence-based approach was adopted for calculating the economic cost of tobacco use in Sri Lanka in 2015. The economic costs of tobacco-related cancers were calculated as direct costs for treating tobacco-related cancers and indirect cost due to productivity loss due to premature mortality and morbidity. The costs of smoking and smokeless tobacco were calculated separately and combined to estimate the total cost due to tobacco and ST-related cancers.

### Types of cancer selected for the study

The costs of lung, lip, oral cavity and pharynx (3rd revision of the International Classification of Diseases for Oncology (ICDO-3): C00–C14 except C07-C08 salivary gland neoplasm) and oesophagus, larynx, stomach, kidney, pancreas, colorectal, liver and bladder cancer were calculated for smoking-associated cancers. Costs of cancer of the lip, oral cavity and pharynx (ICDO-3: C00–C14 except C07-C08) and oesophagus were calculated for ST.

### Calculation of smoking/ST attributable fractions (AF)

The following formula is used for calculating smoking/ST AF:

$$AF=\frac{\sum_{j-1}^{n}P_{j}(RR_{j}-1)}{\sum_{j=1}^{n}P_{j}(RR_{j}-1)+1}$$

where j is the exposure category with baseline exposure or no exposure (j=0), RR(j) is the relative risk at exposure level j compared with no consumption and P(j) is the prevalence of the jth category of exposure.

AFs were calculated for each type of cancer. The AFs derived from the formula, together with the number of deaths and healthcare episodes, were used to estimate the number of healthcare episodes attributable to smoking in this study.

### Calculation of direct cost

Direct healthcare cost included both government and out of pocket (OOPE) expenditures for outpatient and inpatient visits as well as clinic visits. Direct costs for outpatient care took into account frequency of clinic visits for the year and the cost to the government of providing such a service per person. An expert panel consisting of eight senior oncologists, two oncosurgeons, five public health consultants, a surgeon and three consultant physicians and two senior economists was consulted to estimate these costs. When calculating the inpatient care cost, the costs of surgery and pharmaceuticals, survival rates for certain cancers, the average intensive care unit (ICU) treatment days required for specific cancers, the average number of days in hospital were expert views of this panel. These costs reflected the experience of the clinicians in both the government and private sectors as all of them worked in both sectors.

Costs incurred by family members in accompanying the patient on different OPD visits and on entering hospital are included under OOPE by family.

### Calculation of indirect cost

Loss of life or withdrawal from the workforce was calculated, considering the earnings for the period up to retirement based on average earnings adjusted for economic growth, the probabilities of survival and employment. The average earnings were taken from the Household Income and Expenditure Survey (HIES) data of the Department of Census and Statistics. Indirect costs in the form of lost earnings due to premature mortality was calculated using the ‘scenario building method’ based on mean income, incorporating annual growth, weights for probability of survival and employment with the lost earnings gap depending on age of death and assumed age of retirement.

A discount rate of 4.5% was used. Lost daily earnings due to absenteeism was calculated based on average monthly earnings as reported in the HIES.

### Data sources

Published reports of surveys conducted by government which are nationally representative such as the National Cancer Registry, reports from Registrar General’s Department and Medical Statistics Unit of the Ministry of Health were used. Studies carried out by postgraduate students in the areas of community medicine and health economics were utilised in developing the best possible estimates when there was a paucity of data.

The extracted data were further validated through expert group meetings with the agreement of all experts. When the data were unavailable (eg, survival rates for certain cancers, the average number of ICU treatment days for specific cancers), the best estimates were made through the consensus of the experts.

Incidence of cancers among males and females were projected for 2015 based on data from the National Cancer Registry 2009 and cross-checked against Globocan 2012 IARC data base for Sri Lanka^3 11^ which was used to estimate the number of patients with cancer in the year 2015.

The number of deaths from each type of cancer were obtained from the 2010 Indoor Mortality and Morbidity Report, of the Medical Statistics Unit, Ministry of Health, Sri Lanka.^12^

Income data were obtained from Household Income and Expenditure Survey year 2012–2013, of the Department of Census and Statistics, of Sri Lanka.^13^

The OOPE of indoor patients and outpatient department, clinic visits, hospital costs, ward management costs including hospital indoor cost per patient and human resource costs were obtained from a comprehensive study conducted in 2014.^14^

Length of stay in the hospital ward and number of clinic visits per year were obtained from the Statistical Cancer Review 2011 of the Medical Statistic Unit, National Cancer Institute, Sri Lanka.^15^

To calculate the AF for smoking, the prevalence of smoking and ST use were obtained from STEP survey in the year 2015 and RRs were derived from international literature, that is, systematic reviews on related cancers.^4–6 16–20^

## Results

AFs for smoking and ST were calculated based on the RRs obtained through the published meta-analysis studies in the literature (table 1).

**Table 1:** Relative risks (RR) and tobacco smoking and smokeless tobacco (ST) attributable fractions (AF) for different types of cancer

Types of cancer	RR		AF		Source of RR
	Male	Female	Male	Female	
Smoking					
Lip, oral, cavity, pharynx	3.43	3.43	41.42%	0.10%	Gandini *et al* 4
Oesophagus	2.52	2.28	30.67%	0.05%	Gandini *et al*^4^
Stomach	1.74	1.45	17.72%	0.02%	Gandini *et al* 4
Pancreas	1.63	1.73	15.49%	0.03%	Gandini *et al* 4
Larynx	6.98	6.98	63.51%	0.24%	Gandini *et al* 4
Trachea, lung, bronchus	9.87	7.58	72.08%	0.26%	Gandini *et al* 4
Cervix, uterine	–	1.73*	–	0.03%	Zeng *et al* 17
Urinary bladder	2.8	2.73	34.37%	0.07%	Gandini *et al* 4
Kidney and renal pelvis	1.59	1.35	14.65%	0.01%	Gandini *et al* 4
Breast	–	1.6*	16.32%	0.03%	Chen *et al* 18
Liver	1.53	1.7	13.36%	0.03%	Wanshni^19^
Colon and rectum	1.09 and 1.24		6.53%	0.01%	Wang *et al* 20
ST					
Lip, oral, cavity, pharynx	3.43	3.43	41.42%	0.10%	Thomas *et al* 5
Oesophagus	2.52	2.28	30.67%	0.05%	Akhtar^6^

*Passive smoking RR.

### Direct healthcare cost

Table 2 contains the direct healthcare costs of smoking and ST attributable cancers disaggregated by inpatient and outpatient care. Inpatient and outpatient care costs relate to state expenditure on healthcare while OOPE is borne by households.

**Table 2:** Direct costs of tobacco smoking and smokeless tobacco (ST)-related cancers in US$

Type of cancer	Inpatient cost	Outpatient cost	OOPE	Total healthcare cost
Smoking				
Lip, oral cavity, pharynx	5 875 994	297 726	3 920 452	10 094 172
Oesophagus	1 320 233	34 958	950 502	2 305 693
Stomach	333 534	6816	185 328	525 679
Pancreas	269 150	805	41 662	311 618
Larynx	2 337 541	158 792	1 483 735	3 980 068
Trachea, lung, bronchus	6 900 016	73 037	2 881 932	9 854 985
Cervix, uterine	1796	118	1403	3318
Urinary bladder	675 634	41 389	490 614	1 207 638
Kidney and renal pelvis	194 797	3818	80 391	279 006
Breast	49 993	11 445	72 475	133 913
Liver	216 774	993	70 108	287 876
Colorectal	293 352	16 637	180 203	490 193
Total direct cost for smoking	18 468 817	646 536	10 358 806	29 474 159
ST				
Lip, oral, cavity, pharynx	593 885	30 091	396 239	1 020 216
Oesophagus	137 812	3649	99 217	240 678
Total direct cost for ST	7 316 971	337 402	4 954 568	12 608 940

OOPE, out of pocket expenditure.

The total estimated direct healthcare cost is US$29.5 million for smoking and US$12.6 million for ST. Total direct cost accounts for 35% of the total economic cost attributable to tobacco (table 4).

Cancer of the lip, oral cavity and pharynx accounted for the highest direct healthcare cost of smoking while lung cancer was the second highest.

### Indirect costs

As shown in table 3, the indirect cost of tobacco smoking was estimated to be approximately US$54.3 million in 2015. Lung cancer accounted for the highest indirect cost. Premature mortality accounted for US$33.2 million and absenteeism accounted for US$21.2 million.

**Table 3:** Indirect costs of tobacco smoking and smokeless tobacco (ST)-related cancers in US$

Type of cancer	Absenteeism cost	Cost due to premature mortality	Total Indirect cost
Smoking			
Lip, oral, cavity, pharynx	7 637 854	9 837 834	17 475 688
Oesophagus	1 830 603	4 111 458	5 942 062
Stomach	305 345	1 340 760	1 646 106
Pancreas	125 017	469 784	594 802
Larynx	2 653 973	0	2 653 973
Trachea, lung, bronchus	6 533 592	15 777 822	22 311 414
Cervix, uterine	2671	950	3621
Urinary bladder	1 315 914	983 093	2 299 007
Kidney and renal pelvis	155 718	0	155 718
Breast	110 946	12 397	123 343
Liver	158 900	3 02 492	461 392
Colorectal	336 092	332 995	669 087
Total indirect cost for smoking	21 166 628	33 169 587	54 336 215
ST			
Lip, oral, cavity, pharynx	7 861 888	9 602 394	17 464 282
Oesophagus	3 012 494	4 203 925	7 216 419
Total indirect cost for ST	10 874 382	13 806 319	24 680 701

The indirect costs of ST were estimated at approximately US$24.7 million (table 4). Cancers of the lip, oral cavity and pharynx had higher indirect costs than for oesophageal cancer. A premature mortality cost was US$13.8 million, while an absenteeism cost was US$10.9 million for ST.

Indirect cost accounted for 65% of the total economic cost of tobacco-related cancers that were studied.

**Table 4:** Summary of costing analysis in US$ (millions)

Cost categories	Cost of tobacco smoking	Cost of smokeless tobacco	Total cost
Direct cost	29.5	12.6	42.1
Inpatients care cost	18.5	7.3	
Outpatient care cost	0.6	0.3	
Out of pocket expenditure	10.4	5.0	
Indirect cost (cost of productivity loss)	54.3	24.7	79.1
Cost of productivity loss due to Absenteeism	21.2	10.9	
Cost of productivity loss due to premature mortality	33.2	13.8	
Total	83.8	37.3	121.2

Please note that rounding off has resulted in a slight discrepancy on the last row.

## Discussion

Economic cost of tobacco-related cancer in Sri Lanka for the year 2015 was US$121.2 million, which accounted for 16.06% of the US$754.81 million allocated for recurrent expenditure for the state health sector for the year 2015.^21^

Economic cost of tobacco-related cancer was 0.15% of the total GDP (US$82 838.66 million) in the year 2015 in Sri Lanka.^21^

Total tobacco tax revenue for the year 2015 was US$592.7 million, which is 20.4% of our cost estimate for tobacco-related cancers.^21^ However, economic costs of other conditions such as non-communicable diseases should be added to the cost to obtain a fair comparison between the taxes and economic costs.

Indirect costs made up the largest cost component accounting for approximately 65% of the total cost, which is similar to findings in previous studies undertaken on this topic.^22 23^ The results show that tobacco exerts a substantial economic burden on the Sri Lankan population. It is imperative, therefore, that policymakers should consider these estimates in developing and implementing public policies and tobacco control measures.

In this paper, we present only the economic costs of selected tobacco-related cancers. It is well established that non-communicable diseases other than cancers comprises a substantial proportion of burden of tobacco-related diseases. The economic costs of these non-communicable diseases related to tobacco, which include heart disease, cerebrovascular disease, diseases of the respiratory and other systems are not included in the final costs presented. Therefore, these findings related to cancers, if taken alone, will substantially underestimate the total economic costs of tobacco in Sri Lanka. Studies have shown that the cost attributable to cancers ranged from 13% of the total cost of smoking in India^24^ to 25% in Thailand^22^ and 35% in Vietnam.^25^ Given these findings, the total economic cost of tobacco in Sri Lanka for 2015 may fall within the range of US$346.3 million to US$932.3 million.

Although there are published costing studies in other countries, a direct comparison of the results is challenging as different studies include different types of diseases, costing methodologies, healthcare consumption patterns. Given similarities in consumption of tobacco products and the challenges posed by tobacco-related cancers in the South Asian region, this study methodology could also provide a sound framework for costing exercises in other countries in the region. The methodology used in this study could be further expanded in the future to consider impacts such as secondhand smoke and the opportunity cost of spending on tobacco in income constrained poor households.

There are some limitations of this study which should be taken into account when interpreting the results. First, this study involved reviewing data from different Departments and Ministries of the government of Sri Lanka. Therefore, the methodologies used, completeness and the timeliness of the reports differ. Second, when calculating the direct cost, cost of prevention, early detection and management of premalignant stages were not considered.

Third, while secondhand smoking is also related to many health hazards, we have only considered the passive smoking effects for breast and cervical cancers. The cost for providing healthcare in the private sector is not considered in this study. However, it is estimated that about 90% of the patients having malignancies seek treatment from the government sector.

When calculating the AFs, RRs were obtained from the systematic reviews, the local RR is available only for lung cancer. Lastly, psychosocial costs of suffering from cancer incurred by patients and their family members have not been assessed.

## Conclusions

Burden of tobacco smoking and ST-related cancers in Sri Lanka is significant. As shown in this study, the economic costs associated with these diseases are enormous, resulting in negative impacts on both the healthcare system individual families and the country’s economy. Therefore, policymakers should take note of this burden and take immediate and effective steps to control tobacco consumption.

**What this paper adds?**

- The economic costs of cancers related to tobacco is significant in Sri Lanka. As seen in other studies carried out in different parts of the world, the indirect costs are greater than the direct costs.
- Though tobacco use is linked to a large proportion on lung cancers epidemiologically, the largest proportion of the total economic costs of tobacco in Sri Lanka was due to cancers of the lip, oral cavity and pharynx.
- The economic costs of cancers due to smokeless tobacco is a significant component of the total economic costs of tobacco-related cancers. This is a factor that needs consideration in tobacco control policy development in countries where smokeless tobacco use is prevalent.
- The guidelines provided by the WHO to calculate the economic costs of tobacco can be adapted to estimate the economic costs of tobacco even in contexts where there are challenges in obtaining data and can be used in resource-poor settings to estimate these costs. This is important as the tobacco industry uses economic arguments in such countries to prevent implementation of effective tobacco control policies, such as policies that increase tobacco taxes.
